# Exploration of intrinsic brain activity in migraine with and without comorbid depression

**DOI:** 10.1186/s10194-018-0876-9

**Published:** 2018-06-26

**Authors:** Mengmeng Ma, Junran Zhang, Ning Chen, Jian Guo, Yang Zhang, Li He

**Affiliations:** 1 Department of Neurology, West China Hospital, Sichuan University, No. 37, Wainan Guoxue Xiang, Chengdu, 610041 Sichuan China; 2 Department of Radiology, Huaxi MR Research Center (HMRRC), West China Hospital, Sichuan University, Chengdu, China; 3 Department of Medical Information Engineering, School of Electrical Engineering and Information, Sichuan University, Chengdu, China

**Keywords:** Migraine, Psychiatric comorbidity, Depression, Neuroimaging, Amplitude of low frequency fluctuation patterns

## Abstract

**Background:**

Major depressive disorder is a common comorbidity in migraineurs. Depression may affect the progression and prognosis of migraine. Few studies have examined the brain function in migraineurs that may cause this comorbidity. Here, we aimed to explore depression-related abnormalities in the intrinsic brain activity of interictal migraineurs with comorbid depression using resting-state functional magnetic resonance imaging.

**Results:**

Significant main effects of migraine and depression provided evidence that migraine and depression jointly affected the left medial prefrontal cortex, which was thought to be the neural basis of self-referential mental activity in previous studies. Abnormalities in this region may contribute to determining the common symptoms of migraine and depression and even result in comorbidity. Additionally, migraineurs with comorbid depression had different developmental trajectories in the right thalamus and fusiform, which were associated with recognizing, transmitting, controlling and remembering pain and emotion.

**Conclusions:**

Based on our findings, the abnormal mPFC which may contribute to determining the common symptoms in migraine and depression and may be a therapeutic target for migraineurs comorbid depression. The different developmental trajectory in thalamus and fusiform indicates that the comorbidity may arise through a specific mechanism rather than simple superposition of migraine and depression.

## Background

Migraine is often accompanied by emotional dysfunction. The depression comorbidity has high prevalence which is the most common comorbidity in migraineurs. The incidence of depression in migraineurs ranges from 8.6% to 47.9%, according to a meta-analysis of 12 studies [1]. Depression in migraineurs is a significant risk factor for migraine chronification, refractoriness to migraine treatments, overuse of medication, increased migraine-related disability, affective temperament dysregulation and suicidal behaviors which contribute to the psychosocial impairment and altered quality of life [2–6]. However, the depression comorbidity is often overlooked in migraineurs. Therefore, it is imperative to attach importance to this comorbidity to prevent, identify and treat depression in patients with migraine.

The combination of migraine and depression has been associated with smaller brain tissue volume than that in patients with one or neither of these conditions [7]. The brains of migraineurs with comorbid depression differed from patients with migraine only or depression only. Many migraine neuroimaging studies explored alterations of the brain, identified abnormal functions of specific brain regions and speculated that these regions may contribute to determining the depressive symptoms of migraine in migraine without aura [8–11]. However, previous researches have bot clearly determined whether these brain regions differ in migraineurs with depression compared with patients diagnosed with migraine only or depression only. We postulate that migraine and depression exert different effects in brain state, particularly the functions of specific brain regions that might be associated with clinical symptom and the shared etiological risk factors of the migraine-depression comorbidity.

The amplitude of low-frequency fluctuation (ALFF) is a way of measuring regional intrinsic brain activity to explore the pathophysiology underlying neurological and psychiatric diseases [12, 13]. We aimed to explore depression-related abnormalities in the intrinsic brain activity of interictal migraineurs with comorbid depression using resting-state functional magnetic resonance imaging (RS-fMRI) and compare the findings among four groups, including migraineurs with depression (dMIG), migraineurs without depression (ndMIG), patients with major depressive disorder (MDD) and healthy controls (HC).

## Results

### Demographic and neuropsychological characteristics

Subject demographics and clinical characteristics are shown in Table 1. With the exception of the HRSD scores, no significant differences were observed among the four groups (F = 18.494, *P* < 0.001). A post hoc test was applied to the mean 24-HRSD scores of the four groups; higher scores were recorded for in the groups of dMIG and MDD than in the groups of ndMIG or HC (*P* < 0.001).

**Table 1:** Demographic and clinical characteristics of all subjects

	dMIG (*n* = 10)	ndMIG (*n* = 22)	MDD (*n* = 13)	HC (*n* = 27)	*P* value
Age, y, mean (SD)	27.8 (9.25)	33.59 (8.07)	30.92 (9.1)	29.48 (7,18)	0.206^a^
Male	3 (30%)	5 (22.7%)	3 (23%)	10 (37%)	0.685^b^
Education, y, mean (SD)	13.6 (3.41)	19 (3.28)	13.65 (2.93)	16.11 (3.02)	0.531^a^
Clinical characters of migraine					
With/without aura	0/10	0/22	NA	NA	
Duration of migraine, y, mean (SD)	7.2 (5.55)	9.82 (7.14)	NA	NA	0.314^c^
Attack frequency, per month, mean (SD)	8.52 (7.02)	4.5 (3.50)	NA	NA	0.08^c^
Attack duration, h, mean (SD)	18.65 (14.39)	12.75 (16.79)	NA	NA	0.42^c^
VAS score (0–10), mean (SD)	6.55 (1.17)	5.88 (1.31)	NA	NA	0.174^c^
HIT-6, mean (SD)	65.1 (6.26)	60 (5.77)	NA	NA	0.081^c^
MoCA, mean (SD)	27.5 (2.88)	27.82 (1.5)	27.92 (1.66)	30.59 (1.83)	0.620^a^
24-HRSD, mean (SD)	26.9 (6.67)	3 (2.23)	27.85 (6.49)	2.48 (2.12)	< 0.001^a d^
14-HAMA, mean (SD)	6 (1.826)	3.82 (2.59)	5.23 (2.28)	4.19 (2.69)	0.088^a^

^a^*P* value for the age, MoCA and neuropsychological scores distribution in the four groups were obtained using a separate one-way ANCOVA tests. Post-hoc tests were then performed using the t-test

^b^*P* value for the gender distribution and sleep disturbance in the four groups were obtained using a chi-squared test

^c^*P* value for the clinical characters distribution for dMIG and ndMIG group were obtained using two sample t-test

^d^Post-hoc paired comparisons showed significant differences between dMIG versus ndMIG and HC, depression versus ndMIG and HC, *P* < 0.001

*dMIG* migraine with depression, *ndMIG* migraine without depression group, *MDD* major depressive disorder, *HC* health control group, *VAS* visual analogue scale, *HIT-6* Headache Impact Test, *MoCA* Montreal Cognitive Assessment, *24-HRSD* 24-Hamilton Rating Scale for Depression, *14-HAMA* Hamilton Anxiety Rating Scale Values are represented as the mean (standard deviation)

### Significant main effects

**Fig. 1:**
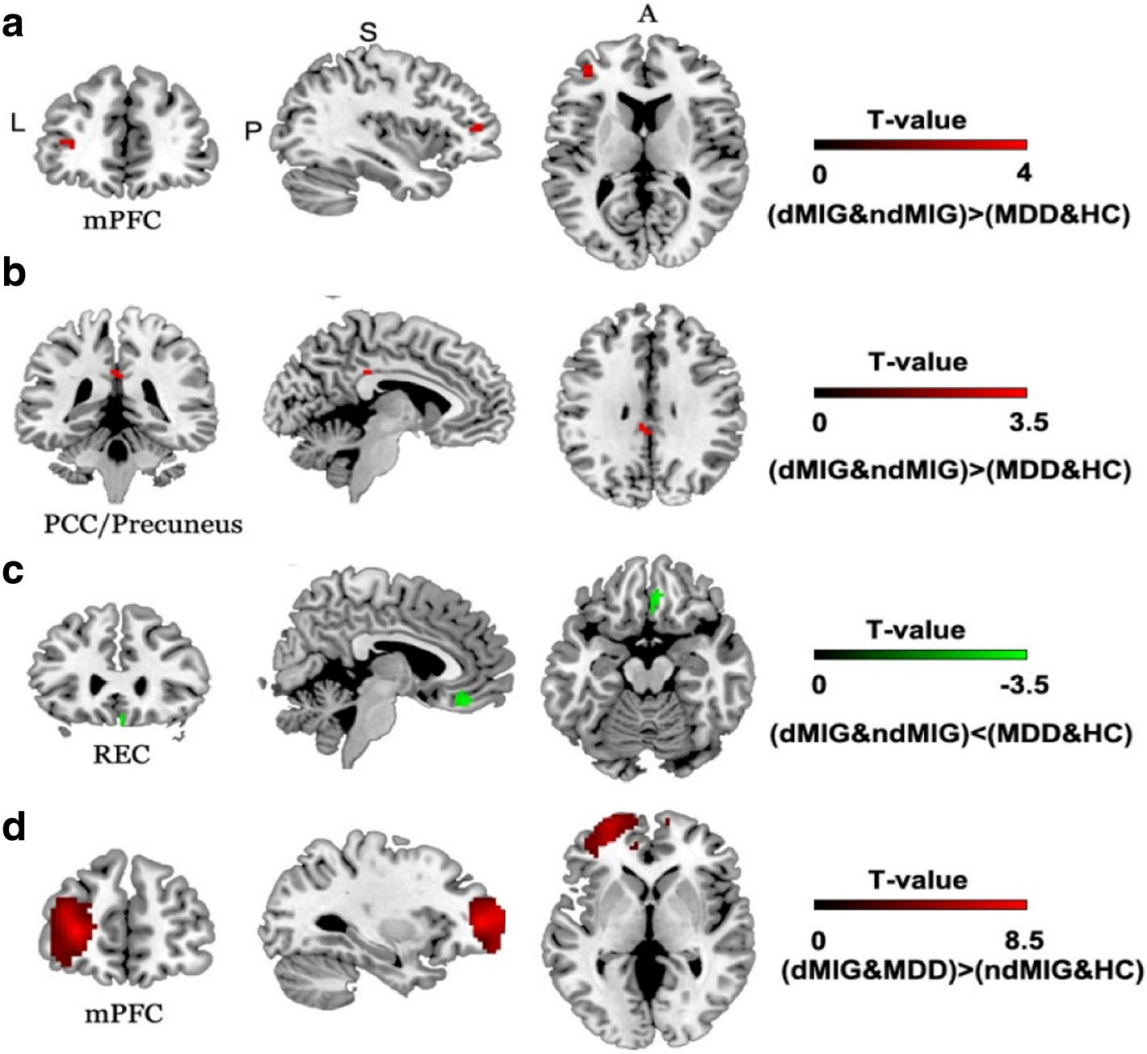
Significant main effect of migraine. Significant main effect of migraine in left medial prefrontal cortex (**a**), the bilateral posterior cingulate cortex/ precuneus (**b**) and the right rectus gyrus (**c**) and main effect of depression in left medial prefrontal cortex (**d**); mPFC: medial prefrontal cortex; REC: rectus gyrus; PCC: posterior cingulate cortex. dMIG: migraineurs with depression; ndMIG: migraineurs without depression; MDD: patients with major depressive disorderand; HC: healthy controls; mPFC: medial prefrontal cortex; PCC: posterior cingulate cortex; REC: rectus gyrus

The two-way ANOVA on ALFF reveled three significant brain regions with a main effect of migraine: the bilateral posterior cingulate cortex/precuneus (PCC/precuneus) (F = 13.10; *P* < 0.001) (Fig. 1a), the right gyrus rectus (REC) (F = 11.83; *P* < 0.001) and left medial prefrontal cortex (mPFC) (F = 16.0; *P* < 0.001) (Fig. 1b). The only region which was observed with a significant main effect of depression was the left mPFC (F = 71.57, *P* < 0.001) (Fig. 1c). The details of these brain regions were shown in the Table 2. Post hoc analysis showed significantly decreased ALFF values in the right REC and increased values in the bilateral PCC/precuneus and left mPFC in dMIG and ndMIG compared with the MDD and HC groups, as well assignificantly increased ALFF values in the left mPFC in dMIG and MDD groups compared with those in ndMIG and HC groups. No significant differences were observed in any other comparison.

**Table 2:** Brain regions showing significant main and interaction effects among four groups

	Region	Hemi	Voxel	BA	Peak voxel MNI coordinates			*T* value
					x	y	z	
Main effect of migraine								
Cluster 1	PCC/Precuneus	L/R	20	23	-3	−36	33	3.62
Cluster 2	REC	R	35	11	6	39	−18	−3.44
Cluster 3	mPFC	L	20	10	−36	45	9	4
Main effect of depression								
Cluster 1	mPFC	L	752	10	−30	51	6	8.46
Interaction effect								
Cluster 1	Thalamus	R	10	NA	12	−12	6	3.30
Cluster 2	fusiform	R	38	37	27	−3	−42	−4.07

*Hemi* hemisphere, *L* left, *R* right, *BA* Brodmann Area, *MNI* Montreal Neurological Institute, x, y, z, coordinates of primary peak locations in the MNI space, *T value* statistical value of peak voxel showing ALFF differences among the four groups, *PCC* posterior cingulated cortex, *REC* rectus gyrus, *mPFC* medial prefrontal cortex

### Interaction effects

**Fig. 2:**
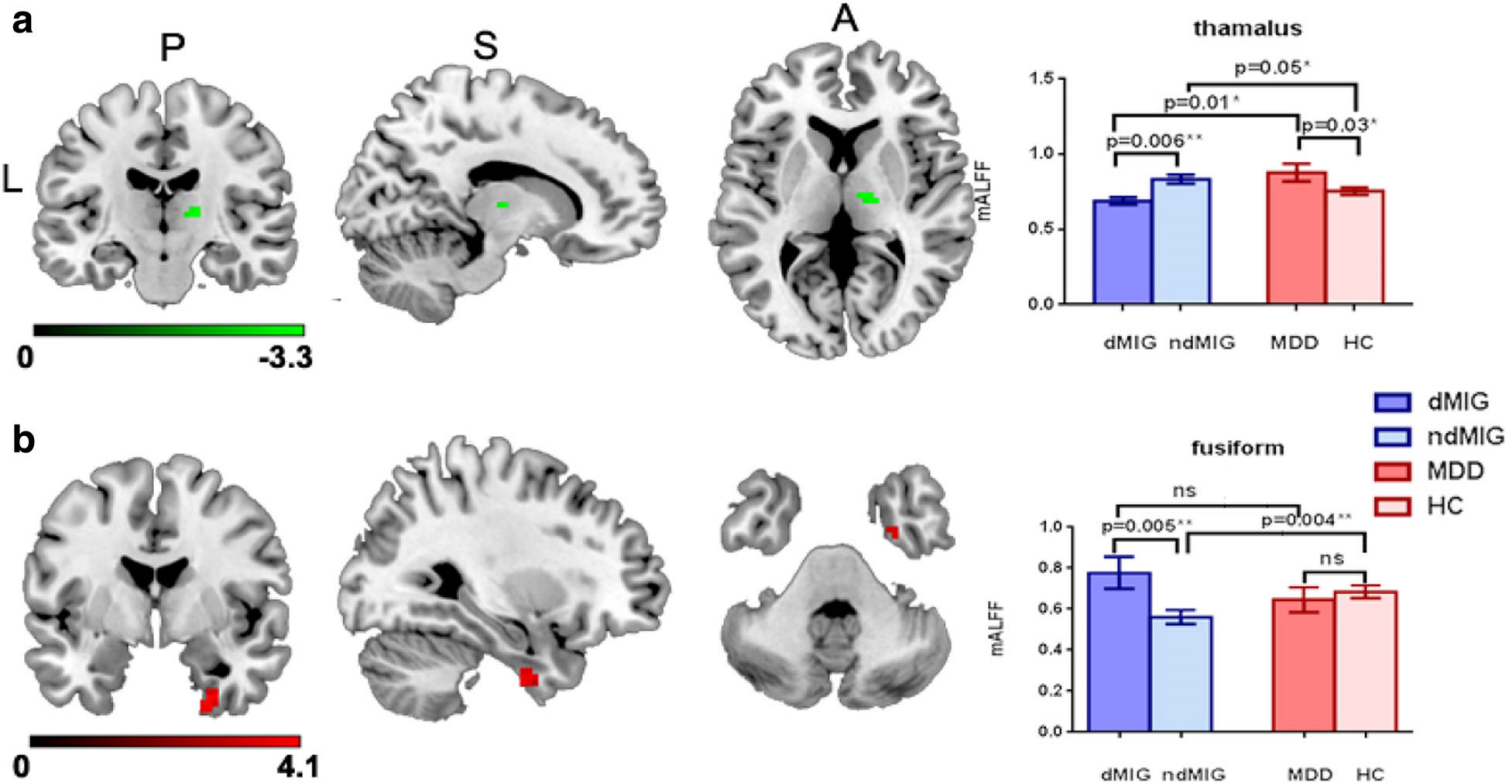
Significant interaction effects. Significant interaction effects in right thalamus (**a**) and the right fusiform (**b**); * uncorrected, *P* < 0.05. ** indicated Bonfornni corrected. *dMIG* migraineurs with depression, *ndMIG* migraineurs without depression, *MDD* patients with major depressive disorder and, *HC* healthy controls

Significant interaction effects were observed in the right thalamus (F = 10.89; *P* < 0.001) (Fig. 2a) and right fusiform (F = 16.56; *P* < 0.001) (Fig. 2b). The details of these brain regions were shown in the Table 2. According to the post hoc analysis, the ALFF values in the right thalamus were decreased in the dMIG group comparing with those in ndMIG group (*P* = 0.006, Bonferroni corrected) and MDD group (*P* = 0.01, Bonferroni corrected). Furthermore, in the fusiform, the dMIG group displayed increased ALFF values compared with those in the ndMIG group (*P* = 0.005, Bonferroni corrected), and the ALFF values of the ndMIG group were decreased compared with those in HC group (*P* = 0.004, Bonferroni corrected), but no difference in any other comparison were observed between the groups.

### Clinical correlations

As these analyses were exploratory, we used a statistical significance level of *P* < 0.01. Significant correlations were observed between the ALFF values for other ROIs and clinical characteristics.

## Discussion

In the current study, we examined the effect of depression on intrinsic brain activity, as reflected by ALFF in migraineurs and HCs among the four groups. Compared with persons without migraine, migraineurs exhibited significantly decreased activity in the right REC and increased intrinsic brain activity in the bilateral PCC/precuneus. Additionally, migraine and depression affected the left mPFC with increasing ALFF simultaneously and exerted different effects in right thalamus and fusiform. Taken together, these findings may yield insights into the comorbidity and provide a basis for developing novel imaging biomarkers for gauging the impact of therapeutics.

Although many studies have shown a clear relationship between migraine and depression, most of them were based on clinical observations, case studies, and genetic epidemiology. Based on these studies, it was speculated that many factors, including serotonergic disorders, hypothalamic-pituitary-adrenal axis hyperactivity, inflammation, and environmental or genetic risk factors for inflammation may converge to result in an altered brain state that could predispose individuals to both migraine and depression [1, 9, 14, 15]. However, the specific changes in brain regions function have not yet been reported in the literature. Therefore, we examined the effect of depression on intrinsic brain activity, reflected by ALFF in migraine and HC among four groups.

Consistent with the findings of previous studies, the current study observed that the depressive patients had increased intrinsic brain activity in the left mPFC. As the anterior node of the default mode network (DMN), the mPFC was highly active at rest but had suppressed activity during cognitive and emotional processing [16, 17]. Converging evidence suggested altered mPFC functional connectivity involved in the development of MDD and the mPFC had long been suspected to be the neural basis of self-referential mental activity [18, 19]. In this study, migraineurs also exhibited increased intrinsic brain activity in the left mPFC. Neuroimaging studies have identified frontal cortical abnormalities in migraineurs [20, 21]. Neuropsychological investigations have highlighted PFC related cognitive impairments in migraineurs, including working memory and executive function deficits [22, 23]. Based on these findings, we speculate that abnormalities in the mPFC region contribute to determining the common symptoms of migraine and depression, even results in the comorbidity and may be a therapeutic target for migraineurs comorbid depression.

In this study, compared with persons without migraine, migraineurs exhibited significantly decreased activity in the right REC and increased intrinsic brain activity in the bilateral PCC/precuneus which has been reported to be dysfunctional in previous studies, particularlythe PCC/precuneus. The REC exhibits a reduced volume in patients with schizophrenia and depression, but relatively fewer studies have reported similar findings in migraine. The REC is part of a circuit that mediates some specific cognitive and emotional functions in humans and plays an important role in the pathogenesis of behavioral addiction combined with substance abuse [24, 25]. Thus, we speculated that the abnormalities in the right REC in migraine may suggest that migraineurs are prone to emotional disorders and drug addiction. The bilateral PCC/ precuneus are the key nodes of DMN and have been observed dysfunction in previous study. There were few reports about activation alteration in migraine. The regions had a high baseline metabolic rate measured by PET. They reduced metabolism and functional connectivity in healthy aging and neurodegenerative diseases like Alzheimier’s disease [26, 27]. However, it was reported that the region increased metabolism and altered functional connectivity in MDD [28, 29]. So we speculated that the altered activity of PCC/ precuneus in migraine might be associated with the depressive tendency. It need more neuroimaging study to verify.Migraineurs with comorbid depression showed decreased intrinsic brain activity in the thalamus compared with ndMIG and MDD and increased activity in the fusiform compared with ndMIG. As a critical multifunctional relay center, the thalamus is considered the transmission control center of emotion and is thought to play an important role in the transmission of nociceptive inputs to cortical structures that are speculated to be involved in the migraine-depression comorbidity, as depression may affect the homeostasis of the transmission of headache-related nociceptive signals from the thalamus to the cortex [30, 31]. Thalamic neurons must adjust to constantly changing physiological (sleep, wakefulness, food intake, body temperature, heart rate, and blood pressure), behavioral (addiction and isolation), cognitive (attention, learning, and memory use), and affective (stress, anxiety, depression, and anger) parameters to maintain homeostasis [32]. The current finding of decreased activity in the thalamus may support the hypothesis that the habituation deficit in migraineurs with depression is due to a reduced preactivation level of sensory cortices and not to increased excitability or reduced intracortical inhibition. Moreover, the ALFF of the right fusiform negatively correlated with the mean headache degree of migraine in ndMIG. Vey fewstudies of fusiform activity have been conducted, and they have found divergent results concerning migraine [33, 34]. The divergence in finding from migraine may be due to differences in the course of migraine or a lack of consideration of the emotional effect. The function of the fusiform has been linked to various neural pathways related to recognition, cognitive pain processing and various neurological phenomena such as synesthesia, dyslexia, and prosopagnosia [35]. Atypical functions and structure of the fusiform gyrus have been identified in chronic low back pain, fibromyalgia, and cluster headache patients [35, 36]. Depression combined with migraine may recruit additional neural resources to improve task performance and enhance the mental imagery of pain based on memories of recurrent migraine headaches, raising the possibility of migraine chronification [37].

Migraineurs with comorbid depression showed significantly decreased intrinsic brain activity in the thalamus and increased activity in the fusiform compared with ndMIG. According to genetic studies, migraine may be a symptom or consequence of MDD in at least a subset of migraine patients with depression as the comorbid depression and migraine were genetically most similar to depression patient [38]. Although our results revealed that migraineurs with comorbid depression exhibit significant changes in brain activity in the thalamus and fusiform gyrus (indicated Bonfornni corrected) with those migraineurs and patients with depression. We speculate that these changes determine the different symptoms. This finding highlights the abnormal developmental patterns of intrinsic brain activity in migraine-depression comorbidity as opposed to migraine or depression alone.

Recently, the functional organization of white matter (WM) in resting-state has received greater attention. Several studies demonstrated the existence of functional brain activity in the WM in normal controls and insufficient or ineffective communication associated with WM abnormalities in many brain disorders, including schizophrenia, epilepsy, Alzheimer’s and Parkinson’s disease [39–43]. In particular, diffusion tensor imaging (DTI) can offer a unique noninvasive insight into the microstructure of WM tracts in the living brain. The DTI studies have revealed abnormal WM integrity in patients with migraine and depression. Solid evidence has been shown that patients with migraine and depression show abnormal diffusion characteristics in several WM tracts such as frontal WM cluster, corpus callosum, optic radiation and internal capsule [44–47]. In current study, it can be found that the partials of the regions of main effect were located in the white matter (WM), especially the with matter in mPFC which has been detected increased radial diffusivity in migraine and depression [45, 48]. Converging the considerable evidence and current results, the mPFC may be the key region of migraineurs cormobid depression.

Based on the results from this study, migraine and depression selectively affect the function of the posterior and anterior nodes of the DMN [49–51]. The DMN is highly related to cognitive processes and influences behavior in response to the environment in a predictive manner [52]. The network reduces its activation during task-related activities or those that require executive function in the healthy human brain. Previous studies have demonstrated that the DMN is dysfunctional in a resting state in various neurological and neuropsychiatric disorders, such as migraine, epilepsy and depression [50, 53, 54]. Studies of functional connectivity and the DMN are necessary to further examine the comorbidity of migraine and depression. Moreover, the relationship between the intrinsic brain activity and cortical thickness has been shown that the spatial distribution of cortical thickness was negatively correlated with surface-based intrinsic brain activity at whole-brain level in epilepsy [55]. This finding contributes to the analysis clinical application of the current results and combine with previous studies of cortical thickness in migraine or depression. But there is no study reporting the cortical thickness in migraine comorbid with depression. Thus, it may be a good direction to research the cortical thickness and elucidate its associate with intrinsic brain activity to aware the brain function of migraineur comorbid with depression.

Similar to most clinical studies, the present study has several limitations. First,, we only included the patients who had never taken any antidepressants or durgs to prevent migraine before the RS-fMRI scan in order to avoid the effects of drugs for migraine and depression on brain activation and the possible withdrawal effect. Therefore, only 10 of 120 migraineurs with depression completed the fMRI scan. The small number of patients could leave our study too underpowered to reveal more subtle findings, such as correlations between clinical features, although our research used rigorous statistical methods with appropriate corrections. Second, as this study employed a cross-sectional design, we were unable to easily estimate whether this abnormal intrinsic brain activity exhibited dynamic changes with follow-up. In the future, it is necessary to conduct prospective longitudinal studies with large sample sizes to assess the associations between migraine with depression and changes in brain activation. An fMRI assessment of changes in functional connectivity might help clarify the association between migraine headache and MDDand elucidate the direction of comorbidity development.

## Conclusions

This study explored intrinsic brain activity in migraine with comorbid depression in four groups. As expected, migraine and depression jointly affected left mPFC, which is thought to be the neural basis of self-referential mental activity and has been shown the increased ALFF in depression patients. We speculated the abnormal mPFC may contribute to determining the common symptoms in migraine and depression and may be a therapeutic target for migraineurs comorbid depression. Besides, it was found that migraine and depression had apparently different developmental trajectory in the right thalamus and right fusiform, which are associated with recognizing, transmitting, controlling and remembering pain and emotion. The abnormalities in these regions may be relevant to the special phenotype of the migraine and depression comorbidity, and our results suggest that the comorbidity arises through a specific mechanism rather than a mere superposition of migraine and depression. In the future, migraine studies may need to consider depression when interpreting fMRI data.

## Methods

### Study population

All the patients were recruited from the Department of Neurology or Psychiatry of West China Hospital between June 2016 and February 2017 and were evaluated by at least two neurologists and two psychiatrists. The diagnosis of migraine without aura was made according to the International Classification of Headache Disorders, 3rd edition (beta version) (ICHD-3 beta). The diagnosis of migraine without aura in ICHD-3 beta is not different from the diagnosis based on ICHD-3 [56]. Depression was diagnosed according to the Diagnostic and Statistical Manual of Mental Disorders, 5th Edition (DSM-5) criteria. The inclusion criteria of all subjects were (1) Han ethnicity (the predominant ethnic group in China), (2) 18–60 years of age, (3) right-handed, and (4) first came to the clinic seeking medical help for migraine and depression. The exclusion criteria were (1) a history of systemic disease, chronic pain disorders and serious neurological disorders; (2) a history of analgesic overuse, as we aimed to avoid recruiting patients with common secondary headache-medication overused headache; (3) the presence of intracranial lesions detected in previous MRI or CT scans; (4) had the contraindications for MRI scanning, including metal implant or psychiatric disorders, such as anxiety (claustrophobia), that prevented patients from completing the MRI scanning; (5) a headache attack during RS-fMRI or within 24 h after scanning.

#### The dMIG group

The subjects were diagnosed with migraine without aura and comorbid depression according to the criteria of ICHD-3 beta and the DSM-5. They were all initially diagnosed with migraine and depression and had not used any antidepressants or drugs to preventing migraine. All the patients in this group had scores of greater than 24 on 24-item Hamilton Rating Scale for Depression (24-HRSD). The patients had no history of other types of headache, anxiety or other psychiatric disorders.

#### The ndMIG group

The patients in this group were recruited from the Department of Neurology and diagnosed with migraine without aura and had no history of any psychiatric disorders including MDD or depressive mood. The patients were all initially diagnosed with migraine and had not previously used any drugs to prevent migraine. All the patients in this group had scores of less than 8 on the 24-HRSD.

#### The MDD group

The patients in the MDD group were diagnosed according to the DSM-5 by at least two psychiatrists. They had no history of migraine, other types of headache, anxiety or other psychiatric disorders. The patients were all first diagnosed with depression and had never used any antidepressant.

#### The HC group

HC were recruited in June 2016 and February 2017. They were evaluated by two neurologists and two psychiatrists and had no history of migraine, depression, alcohol dependence or of using medications. Besides, they had no family history of migraine and psychiatric disorders.

The subjects in the four groups were matched for sex, mean age, and years of education. After evaluating the potential subjects, we established 4 groups, including 10 dMIG, 22 ndMIG (migraineurs with no depressive mood), 13 MDD and 27 HC. The demographic data of all the subjects and clinical characteristics of migraineurs were obtained, including age, gender, years of education, migraine duration, attack frequency and attack duration of migraine, headache degree, the Headache Impact Test-6 (HIT-6) and scores on the 24-HRSD, 14-Hamilton Anxiety Rating Scale (14-HAMA) and Montreal Cognitive Assessment (MoCA).

### MRI data acquisition

All the scans were performed on a 3.0-T MRI scanning system (Siemens Trio Tim, Erlangen, Germany) at the Department of Radiology, West China Hospital of Sichuan University. Earplugs and tight padded clamps were used to minimize noise exposure and head motion. The participants were instructed to remain still, close their eyes, remain awake and let their minds wander. Scanning was terminated if the participant complained of any discomfort. Images of structures were obtained using routine T1 weighted imaging, and the RS-fMRI was conducted using an echo-planar imaging (EPI) sequence (TR 2000 ms, TE 30 ms, voxel size 3.75 × 3.75 × 5 mm3, flip angle 90°, slice thickness 5 mm, matrix 64 × 64, FOV 24 × 24 cm^2^). Each resting-state scan lasted for 6 min and 180 volumes were collected. Two experienced neuroradiologists performed all scan and checked the images to exclude brain tissue abnormalities in the four groups.

### RS-fMRI image preprocessing

Functional images were preprocessed with the software Data Processing Assistant for Resting-State fMRI, Advanced Edition (DPARSF A, http://rfmri.org/DPARSF) in MATLAB (R2010b). The first 10 volumes were removed for each subject. The remaining images were corrected by slicing time and realigned. No subjects displayed head movement i.e., head motions exceeding 2.0 mm of translation or 2.0 degrees of rotation during the scanning process to disqualify them from the study. The subsequent processing steps included normalizing the scans into the standard stereotactic space using the Montreal Neurological Institute EPI template; smoothing with an 8 × 8 × 8 mm^3^ full width at half-maximum kernel; and removing covariates by linear regression, including head motion parameters, averaged signal from the white matter and signal from the cerebrospinal fluid, to further reduce the effects of confounding factors. Finally, the functional images were detrended by bandpass filtering (0.01–0.08 Hz) to reduce low-frequency drift and physiological high-frequency respiratory and cardiac noise.

### ALFF analysis

We computed the ALFF value for each voxel using DPARSF software (Advanced Edition, http://rfmri.org/DPARSF) to construct the intrinsic brain activity map of each subject, The power spectrum was obtained after the time series of each voxel was transformed to the frequency domain. The average square root of the power spectrum was regarded as the ALFF. For the purpose of reducing the global effects of variability across subjects, the ALFF value of each voxel was normalized to the global mean ALFF value [49].

### Statistical analysis

The values are reported as absolute numbers with percentages for categorical variables and as means with SDs for continuous variables. χ2 tests, t-tests and analysis of variance (ANOVA) were performedto compare categorical variables between groups, continuous variables between two groups, and continuous variables among four groups, respectively. The level of statistical significance was defined as combined *P* < 0.01.

Two-way ANOVA with migraine and depression as between-subject factors was performed on the individual normalized ALFF maps using statistical parametric mapping (SPM 8, www.fil.ion.ucl.ac.uk/spm). Comparisons of the main effects and interaction effect were corrected using AlphaSim correction. The level of statistical significance was defined as combined *P* < 0.05 (combined height threshold *P* < 0.001 and a minimum cluster size of 10 voxels with AlphaSim correction).The brain regions with significant main effects and interaction effects were designated as regions of interest (ROIs). Post hoc tests were then performed on the ROIs using Bonferroni correction (*P* < 0.05/4 = 0.0125). We computed Pearson correlation coefficients of average ROI ALFF values versus clinical factors of migraine (duration, mean attack frequency, duration of daily attack, mean visual analogue scale score (VAS score) and HIT-6) and neuropsychological variables (MoCA, 14-HRSD and 14-HAMA) separately for dMIG, ndMIG and MDD groups to explore the relationship between intrinsic brain activity and the clinical characteristics.

## Abbreviations

14-HAMA: 14-item Hamilton Anxiety Rating Scale
24-HRSD: 24-item Hamilton Rating Scale for Depression
ALFF: the amplitude of low-frequency fluctuation
ANOVA: Analysis of variance
dMIG: Migraineurs with depression
DMN: Default mode network
DPARSF A: Data Processing Assistant for Resting-State fMRI, Advanced Edition
DSM-5: The Diagnostic and Statistical Manual of Mental Disorders, 5th edition
EPI: Echo-planar imaging
HC: Healthy controls
HIT-6: Headache Impact Test-6
ICHD− 3: The International Classification of Headache Disorders, 3-rd
MDD: Major depressive disorder
mFPC: Medial prefrontal cortex
MoCA: Montreal Cognitive Assessment
ndMIG: Migraineurs without depression
PCC: Posterior cingulate cortex
REC: Right gyrus rectus
ROIs: Regions of interest
RS-fMRI: Resting-state functional magnetic resonance imaging
SPM 8: Statistical parametric mapping 8

## References

[1] Antonaci F, Nappi G, Galli F, Manzoni GC, Calabresi P, Costa A (2011). Migraine and psychiatric comorbidity: a review of clinical findings. J Headache Pain.

[2] Ashina S, Serrano D, Lipton RB, Maizels M, Manack AN, Turkel CC, Reed ML, Buse DC (2012). Depression and risk of transformation of episodic to chronic migraine. J Headache Pain.

[3] Lantéri-Minet M, Radat F, Chautard MH, Lucas C (2005). Anxiety and depression associated with migraine: influence on migraine subjects’ disability and quality of life, and acute migraine management. Pain.

[4] Peck KR, Smitherman TA, Baskin SM (2015). Traditional and alternative treatments for depression: implications for migraine management. Headache.

[5] Radat F, Creac'h C, Swendsen JD, Lafittau M, Irachabal S, Dousset V, Henry P (2005). Psychiatric comorbidity in the evolution from migraine to medication overuse headache. Cephalalgia.

[6] Serafini G, Pompili M, Innamorati M, Gentile G, Borro M, Lamis DA, Lala N, Negro A, Simmaco M, Girardi P, Martelletti P (2012). Gene variants with suicidal risk in a sample of subjects with chronic migraine and affective temperamental dysregulation. Eur Rev Med Pharmacol Sci.

[7] Gudmundsson LS, Scher AI, Sigurdsson S, Geerlings MI, Vidal JS, Eiriksdottir G, Garcia MI, Harris TB, Kjartansson O, Aspelund T, van Buchem MA, Gudnason V, Launer LJ (2013). Migraine, depression, and brain volume: the AGES-Reykjavik study. Neurology.

[8] Li XL, Fang YN, Gao QC, Lin EJ, Hu SH, Ren L, Ding MH, Luo BN (2011). A diffusion tensor magnetic resonance imaging study of corpus callosum from adult patients with migraine complicated with depressive/anxious disorder. Headache.

[9] Tietjen GE, Buse DC, Fanning KM, Serrano D, Reed ML, Lipton RB (2015). Recalled maltreatment, migraine, and tension-type headache: results of the AMPP study. Neurology.

[10] Valfrè W, Rainero I, Bergui M, Pinessi L (2008). Voxel-based morphometry reveals gray matter abnormalities in migraine. Headache.

[11] Xue T, Yuan K, Zhao L, Yu D, Zhao L, Dong T, Cheng P, von DKM, Qin W, Tian J (2012). Intrinsic brain network abnormalities in migraines without aura revealed in resting-state fMRI. PLoS One.

[12] Duff EP, Johnston LA, Xiong J, Fox PT, Mareels I, Egan GF (2008). The power of spectral density analysis for mapping endogenous BOLD signal fluctuations. Hum Brain Mapp.

[13] Fox MD, Raichle ME (2007). Spontaneous fluctuations in brain activity observed with functional magnetic resonance imaging. Nat Rev Neurosci.

[14] Lipton RB, Silberstein SD (1994). Why study the comorbidity of migraine. Neurology.

[15] Minen MT, De Dhaem OB, Van Diest AK, Powers S, Schwedt TJ, Lipton R, Silbersweig D (2016). Migraine and its psychiatric comorbidities. J Neurol Neurosurg Psychiatry.

[16] Lui S, Wu Q, Qiu L, Yang X, Kuang W, Chan RC, Huang X, Kemp GJ, Mechelli A, Gong Q (2011). Resting-state functional connectivity in treatment-resistant depression. Am J Psychiatry.

[17] Greicius MD, Flores BH, Menon V, Glover GH, Solvason HB, Kenna H, Reiss AL, Schatzberg AF (2007). Resting-state functional connectivity in major depression: abnormally increased contributions from subgenual cingulate cortex and thalamus. Biol Psychiatry.

[18] Ferenczi EA, Zalocusky KA, Liston C, Grosenick L, Warden MR, Amatya D, Katovich K, Mehta H, Patenaude B, Ramakrishnan C, Kalanithi P, Etkin A, Knutson B, Glover GH, Deisseroth K (2016). Prefrontal cortical regulation of brainwide circuit dynamics and reward-related behavior. Science.

[19] Liu J, Ren L, Womer FY, Wang J, Fan G, Jiang W, Blumberg HP, Tang Y, Xu K, Wang F (2014). Alterations in amplitude of low frequency fluctuation in treatment-naïve major depressive disorder measured with resting-state fMRI. Hum Brain Mapp.

[20] Bender S, Oelkers-Ax R, Resch F, Weisbrod M (2006). Frontal lobe involvement in the processing of meaningful auditory stimuli develops during childhood and adolescence. Neuroimage.

[21] Rocca MA, Ceccarelli A, Falini A, Tortorella P, Colombo B, Pagani E, Comi G, Scotti G, Filippi M (2006). Diffusion tensor magnetic resonance imaging at 3.0 tesla shows subtle cerebral grey matter abnormalities in patients with migraine. J Neurol Neurosurg Psychiatry.

[22] Schmitz N, Arkink EB, Mulder M, Rubia K, Admiraal-Behloul F, Schoonman GG, Kruit MC, Ferrari MD, van Buchem MA (2008). Frontal lobe structure and executive function in migraine patients. Neurosci Lett.

[23] Shallice T, Burgess PW (1991). Deficits in strategy application following frontal lobe damage in man. Brain.

[24] Andreasen NC, O'Leary DS, Cizadlo T, Arndt S, Rezai K, Watkins GL, Ponto LL, Hichwa RD (1995). Remembering the past: two facets of episodic memory explored with positron emission tomography. Am J Psychiatry.

[25] Chen X, Wang Y, Zhou Y, Sun Y, Ding W, Zhuang Z, Xu J, Du Y (2014). Different resting-state functional connectivity alterations in smokers and nonsmokers with internet gaming addiction. Biomed Res Int.

[26] Leech R, Sharp DJ (2014). The role of the posterior cingulate cortex in cognition and disease. Brain.

[27] Irish M, Halena S, Kamminga J, Tu S, Hornberger M, Hodges JR (2015). Scene construction impairments in Alzheimer’s disease - a unique role for the posterior cingulate cortex. Cortex.

[28] Serra-Blasco M, de Vita S, Rodríguez MR, de Diego-Adeliño J, Puigdemont D, Martín-Blanco A, Pérez-Egea R, Molet J, Álvarez E, Pérez V, Portella MJ (2015). Cognitive functioning after deep brain stimulation in subcallosal cingulate gyrus for treatment-resistant depression: an exploratory study. Psychiatry Res.

[29] Lozano AM, Mayberg HS, Giacobbe P, Hamani C, Craddock RC, Kennedy SH (2008). Subcallosal cingulate gyrus deep brain stimulation for treatment-resistant depression. Biol Psychiatry.

[30] Kupers R, Kehlet H (2006). Brain imaging of clinical pain states: a critical review and strategies for future studies. Lancet Neurol.

[31] Noseda R, Kainz V, Borsook D, Burstein R (2014). Neurochemical pathways that converge on thalamic trigeminovascular neurons: potential substrate for modulation of migraine by sleep, food intake, stress and anxiety. PLoS One.

[32] Noseda R, Borsook D, Burstein R (2017). Neuropeptides and neurotransmitters that modulate Thalamo-cortical pathways relevant to migraine headache. Headache.

[33] Schwedt TJ, Chong CD, Chiang CC, Baxter L, Schlaggar BL, Dodick DW (2014). Enhanced pain-induced activity of pain-processing regions in a case-control study of episodic migraine. Cephalalgia.

[34] Wang JJ, Chen X, Sah SK, Zeng C, Li YM, Li N, Liu MQ, Du SL (2016). Amplitude of low-frequency fluctuation (ALFF) and fractional ALFF in migraine patients: a resting-state functional MRI study. Clin Radiol.

[35] Glass JM, Williams DA, Fernandez-Sanchez ML, Kairys A, Barjola P, Heitzeg MM, Clauw DJ, Schmidt-Wilcke T (2011). Executive function in chronic pain patients and healthy controls: different cortical activation during response inhibition in fibromyalgia. J Pain.

[36] Yang FC, Chou KH, Fuh JL, Huang CC, Lirng JF, Lin YY, Lin CP, Wang SJ (2013). Altered gray matter volume in the frontal pain modulation network in patients with cluster headache. Pain.

[37] Yetkin FZ, Rosenberg RN, Weiner MF, Purdy PD, Cullum CM (2006). FMRI of working memory in patients with mild cognitive impairment and probable Alzheimer's disease. Eur Radiol.

[38] Ligthart L, Hottenga JJ, Lewis CM, Farmer AE, Craig IW, Breen G, Willemsen G, Vink JM, Middeldorp CM, Byrne EM, Heath AC, Madden PA, Pergadia ML, Montgomery GW, Martin NG, Penninx BW, McGuffin P, Boomsma DI, Nyholt DR (2014). Genetic risk score analysis indicates migraine with and without comorbid depression are genetically different disorders. Hum Genet.

[39] Ji GJ, Liao W, Chen FF, Zhang L, Wang K (2017). Low-frequency blood oxygen level-dependent fluctuations in the brain white matter: more than just noise. Sci Bull (Beijing).

[40] Bohnen NI, Albin RL (2011). White matter lesions in Parkinson disease. Nat Rev Neurol.

[41] Caso F, Agosta F, Mattavelli D, Migliaccio R, Canu E, Magnani G, Marcone A, Copetti M, Falautano M, Comi G, Falini A, Filippi M (2015). White matter degeneration in atypical Alzheimer disease. Radiology.

[42] Dong D, Wang Y, Chang X, Jiang Y, Klugah-Brown B, Luo C, Yao D (2017). Shared abnormality of white matter integrity in schizophrenia and bipolar disorder: a comparative voxel-based meta-analysis. Schizophr Res.

[43] Xue K, Luo C, Zhang D, Yang T, Li J, Gong D, Chen L, Medina YI, Gotman J, Zhou D, Yao D (2014). Diffusion tensor tractography reveals disrupted structural connectivity in childhood absence epilepsy. Epilepsy Res.

[44] Rocca MA, Colombo B, Pagani E, Falini A, Codella M, Scotti G, Comi G, Filippi M (2003). Evidence for cortical functional changes in patients with migraine and white matter abnormalities on conventional and diffusion tensor magnetic resonance imaging. Stroke.

[45] Szabó N, Kincses ZT, Párdutz A, Tajti J, Szok D, Tuka B, Király A, Babos M, Vörös E, Bomboi G, Orzi F, Vécsei L (2012). White matter microstructural alterations in migraine: a diffusion-weighted MRI study. Pain.

[46] Nobuhara K, Okugawa G, Sugimoto T, Minami T, Tamagaki C, Takase K, Saito Y, Sawada S, Kinoshita T (2006). Frontal white matter anisotropy and symptom severity of late-life depression: a magnetic resonance diffusion tensor imaging study. J Neurol Neurosurg Psychiatry.

[47] Mettenburg JM, Benzinger TL, Shimony JS, Snyder AZ, Sheline YI (2012). Diminished performance on neuropsychological testing in late life depression is correlated with microstructural white matter abnormalities. Neuroimage.

[48] Li L, Ma N, Li Z, Tan L, Liu J, Gong G, Shu N, He Z, Jiang T, Xu L (2007). Prefrontal white matter abnormalities in young adult with major depressive disorder: a diffusion tensor imaging study. Brain Res.

[49] Zang YF, He Y, Zhu CZ, Cao QJ, Sui MQ, Liang M, Tian LX, Jiang TZ, Wang YF (2007). Altered baseline brain activity in children with ADHD revealed by resting-state functional MRI. Brain and Development.

[50] Sheline YI, Barch DM, Price JL, Rundle MM, Vaishnavi SN, Snyder AZ, Mintun MA, Wang S, Coalson RS, Raichle ME (2009). The default mode network and self-referential processes in depression. Proc Natl Acad Sci U S A.

[51] Zhang J, Su J, Wang M, Zhao Y, Yao Q, Zhang Q, Lu H, Zhang H, Wang S, Li GF, Wu YL, Liu FD, Shi YH, Li J, Liu JR, Du X (2016). Increased default mode network connectivity and increased regional homogeneity in migraineurs without aura. J Headache Pain.

[52] Hamilton JP, Farmer M, Fogelman P, Gotlib IH (2015). Depressive rumination, the default-mode network, and the dark matter of clinical neuroscience. Biol Psychiatry.

[53] Hsiao FJ, Yu HY, Chen WT, Kwan SY, Chen C, Yen DJ, Yiu CH, Shih YH, Lin YY (2015). Increased intrinsic connectivity of the default mode network in temporal lobe epilepsy: evidence from resting-state MEG recordings. PLoS One.

[54] Tessitore A, Russo A, Giordano A, Conte F, Corbo D, De Stefano M, Cirillo S, Cirillo M, Esposito F, Tedeschi G (2013). Disrupted default mode network connectivity in migraine without aura. J Headache Pain.

[55] Liao W, Wang J, Xu T, Zhang ZQ, Ji GJ, Xu Q, Wang ZG, Yang F, Zuo XN, Qiu AQ, Zang YF, Lu GM, Chen HF (2016). Altered relationship between thickness and intrinsic activity amplitude in generalized tonic–clonic seizures. Scirnce Bulletin.

[56] Headache classification Committee of the International Headache Society (IHS) (2018). The international classification of headache disorders, 3rd edition. Cephalalgia.

